# *In Vitro* pH Activity of Ibrexafungerp against Fluconazole-Susceptible and -Resistant *Candida* Isolates from Women with Vulvovaginal Candidiasis

**DOI:** 10.1128/AAC.00562-21

**Published:** 2021-07-16

**Authors:** Jack D. Sobel, Katyna Borroto-Esoda, Nkechi Azie, David Angulo

**Affiliations:** a Wayne State University School of Medicine, Detroit, Michigan, USA; b SCYNEXIS, Inc., Jersey City, New Jersey, USA

**Keywords:** candidiasis, ibrexafungerp, pH, vulvovaginal

## Abstract

The vaginal environment with candidiasis has a pH of 3.8 to 4.5 and this has a negative effect on the activity of antifungals. Ibrexafungerp was evaluated against 187 *Candida* isolates, including fluconazole-sensitive and -resistant Candida albicans, Candida glabrata, Candida krusei, Candida parapsilosis, and Candida tropicalis with the media adjusted to pH 7.0 and pH 4.5. Ibrexafungerp MIC values were not adversely affected when tested at pH 4.5. Ibrexafungerp exhibited significant activity against all isolates at pH 4.5.

## INTRODUCTION

Vulvovaginal candidiasis (VVC) is a common fungal infection that affects up to 8 in 10 women in their lifetime, with 40% to 45% experiencing two or more episodes (1, 2). An analysis of direct health care costs of noninvasive candidiasis in the United States reported that 1.4 million outpatient visits occur annually due to VVC at an estimated cost of $368 million (3, 4). Between 85% and 95% of cultured yeast species causing VVC are Candida albicans, but recent reports suggest an increased prevalence of non-*albicans Candida* (NAC) in about 10% to 30% of patients with VVC (5, 6), with the prevalence of Candida glabrata increasing (7). NAC species are frequently, but not always, resistant to azole agents (7, 8). Vaginal NAC species include C. glabrata, C. krusei, C. parapsilosis, C. lusitaniae, C. famata, C. tropicalis, and C. dubliniensis (7). The increased recent incidence of non-*albicans Candida* is thought to be due to overuse of azole antifungal vaginal products, single-dose treatments, and low-dosage azole maintenance regimens (5, 7, 9). Recent studies further report a rapidly growing incidence of resistance of C. albicans to the azole drug class (10).

Multiple previous *in vitro* studies have shown significantly reduced activity of fluconazole in low-pH testing environments, which is of concern given the vaginal pH found in women with VVC is invariably <4.5 (11–13). Antifungal agents selected for treating VVC should provide activity against all *Candida* spp. at a vaginal pH of 4 to 4.5 (14).

Ibrexafungerp is an oral antifungal agent belonging to a novel class of glucan synthase inhibitors, triterpenoids, that have broad *in vitro* activity against *Candida*, including azole-resistant *Candida* species (15, 16). Ibrexafungerp is fungicidal against *Candida* spp. (17) and has demonstrated high penetration in vaginal tissue is preclinical models (18). Ibrexafungerp is being developed for the treatment of vulvovaginal candidiasis, as well as systemic fungal infections due to resistant isolates. This study evaluated the effect of low-pH environments on the *in vitro* activity of ibrexafungerp against clinical vulvovaginal isolates, including isolates resistant to fluconazole.

Ibrexafungerp was evaluated *in vitro* against 187 vaginal *Candida* isolates obtained from women with VVC who were treated at the Wayne State University Vaginitis Clinic. This included 52 fluconazole-resistant C. albicans (FLU MIC >2 μg/ml), 30 fluconazole-sensitive C. albicans (FLU MIC ≤2 μg/ml), 30 randomly selected C. glabrata isolates, and 25 each randomly selected isolates of C. krusei, C. parapsilosis, and C. tropicalis. All isolates tested were original clinical vaginal isolates obtained at the Wayne State University Vaginitis Clinic. Identification was achieved by germ tube formation testing, CHROMagar plating, and standard fermentation profile analysis. Isolates were stored in −70°C freezers.

Vaginal isolates were plated on CHROMagar to verify purity of culture. These plates were incubated for 48 h at 37°C in ambient air. Susceptibility testing was performed with ibrexafungerp concentrations of 0.03 to 2 μg/ml using a broth microdilution method according to the Clinical and Laboratory Standards Institute (CLSI) document M27-A4 (19) guidelines utilizing pH 7. A 0.1-ml yeast inoculum of 1.5 (±1.0) × 10^3^ cells/ml in RPMI 1640 medium was added to each microdilution well. The trays were then incubated at 35°C for 48 h in ambient air. The MICs were read visually as the lowest antifungal concentration with substantially lower turbidity (80% growth reduction) compared to growth in the antifungal-free growth well for all agents. Testing known ATCC strains of Candida parapsilosis ATCC 22019 and Candida krusei ATCC 6258 ensured quality control. Susceptibility tests were performed simultaneously, with the media adjusted to pH 7.0 with NaOH and pH 4.5 with HCl. Ibrexafungerp MIC readings were conducted at 24 h.

Ibrexafungerp demonstrated *in vitro* activity against all the clinical isolates tested at normal pH 7.0 (Table 1). No differences were observed in ibrexafungerp MIC_90_ values (24 h endpoint at pH 7) between the fluconazole-resistant and fluconazole-sensitive C. albicans isolates (MIC_90_ = 0.03 μg/ml). Against C. glabrata, C. krusei, C. parapsilosis, and C. tropicalis isolates, ibrexafungerp MIC_90_ values were 0.25, 0.5, 0.5, and 0.25 μg/ml, respectively.

**TABLE 1:** Ibrexafungerp effectiveness (MIC in μg/ml) at a pH of 7.0 at 24 h

Parameter	Fluconazole-resistant C. albicans (*n* = 52)	Fluconazole-sensitive C. albicans (*n* = 30)	C. glabrata (*n* = 25)	C. krusei (*n* = 25)	C. parapsilosis (*n* = 25)	C. tropicalis (*n* = 25)
MIC_50_ ibrexafungerp	0.03	0.03	0.125	0.5	0.25	0.125
MIC_90_ ibrexafungerp	0.03	0.03	0.25	0.5	0.5	0.25
Median	0.03	0.03	0.125	0.5	0.25	0.25
Range	0.03–0.06	0.03–0.06	0.06–0.25	0.25–1	0.125–4	0.03–0.25

Ibrexafungerp MIC values were not adversely affected when tested at the lower pH 4.5 (Table 2). No differences were observed in ibrexafungerp’s MIC_90_ values (24 h endpoint at pH 4.5) between the fluconazole-resistant and fluconazole-sensitive C. albicans isolates (MIC_90_ = 0.06 μg/ml). Against C. glabrata, C. krusei, C. parapsilosis, and C. tropicalis isolates, ibrexafungerp MIC_90_ values were 0.5, 0.25, 0.25, and 0.25 μg/ml, respectively.

**TABLE 2:** Ibrexafungerp effectiveness (MIC in μg/ml) at a pH of 4.5 at 24 h

Parameter	Fluconazole-resistant C. albicans (*n* = 52)	Fluconazole-sensitive C. albicans (*n* = 30)	C. glabrata (*n* = 25)	C. krusei (*n* = 25)	C. parapsilosis (*n* = 25)	C. tropicalis (*n* = 25)
MIC_50_ ibrexafungerp	0.03	0.03	0.25	0.25	0.125	0.125
MIC_90_ ibrexafungerp	0.06	0.06	0.5	0.25	0.25	0.25
Median	0.03	0.03	0.25	0.25	0.125	0.25
Range	0.03–0.06	0.03–0.06	0.125–0.5	0.06–0.5	0.03–0.5	0.03–0.5

It is not the standard of clinical care to perform susceptibility tests on all culture-verified clinical isolates of the various *Candida* spp. obtained from symptomatic women with acute VVC. *In vitro* susceptibility tests should be obtained in the presence of refractory or highly frequent recurrent VVC, especially when caused by non-*albicans Candida* spp. Similarly, it is not the practice for commercial or other laboratories to perform testing at any other pH but 7.0, given that the pH of blood is around 7.0, unlike the vagina that normally has an acidic pH of 3.8 to 4.5. Multiple studies have shown that testing at pH 7.0 dramatically underestimates the frequency and reliability of detecting azole-resistant *Candida* isolates that may be resistant to azole therapy and affect azole treatment outcomes, especially C. glabrata, which is particularly prone to the effects of the acidic pH environment of the vagina in women with VVC (11–13, 20–22). Ibrexafungerp exhibited significant *in vitro* activity against all fluconazole-resistant and fluconazole-sensitive vaginal *Candida* species isolates at pH 7.0. The potent *in vitro* activity of ibrexafungerp was retained at a pH of 4.5, relevant for the vaginal milieu, directed at all *Candida* spp. tested. In contrast to other antifungal classes, the *in vitro* susceptibility of ibrexafungerp appears to be unaffected by the pH of the testing medium studied, which provides increased confidence in the potential clinical efficacy of ibrexafungerp for treating VVC.
